# Acute Q fever in febrile patients in northwestern of Iran

**DOI:** 10.1371/journal.pntd.0005535

**Published:** 2017-04-10

**Authors:** Saber Esmaeili, Farhad Golzar, Erfan Ayubi, Behrooz Naghili, Ehsan Mostafavi

**Affiliations:** 1 Research Centre for Emerging and Reemerging Infectious Diseases, National Reference Laboratory for Plague, Tularemia and Q Fever, Pasteur Institute of Iran, Akanlu, Kabudar-Ahang, Hamadan, Iran; 2 Department of Epidemiology and Biostatistics, Pasteur Institute of Iran, Tehran, Iran; 3 Department of Bacteriology, Faculty of Medical Sciences, Tarbiat Modares University, Tehran, Iran; 4 Institute of Natural and Mathematical Science, Massey University, Auckland, New Zealand; 5 Department of Epidemiology and Biostatistics, School of Public Health and Health Research Institute, Tehran University of Medical Sciences, Tehran, Iran; 6 Research Center of Infectious Diseases and Tropical Medicine, Faculty of Medicine, Tabriz university of Medical Science, Tabriz, Iran; University of California San Diego School of Medicine, UNITED STATES

## Abstract

**Background:**

Q fever is an endemic disease in different parts of Iran. This study aimed to investigate the prevalence of acute Q fever disease among at-risk individuals in northwestern Iran.

**Methodology:**

An etiological study was carried out in 2013 in Tabriz County. A total of 116 individuals who were in contact with livestock and had a nonspecific febrile illness were enrolled in the study. IgG phase II antibodies against *Coxiella burnetii* were detected using ELISA.

**Principal findings:**

The prevalence of acute Q fever was 13.8% (95% confidence interval [CI]: 8.0, 21.0%). Headache (87.5%) and fatigue and weakness (81.3%) were the dominant clinical characteristics among patients whit acute Q fever. Acute lower respiratory tract infection and chills were poorly associated with acute Q fever. Furthermore, 32% (95% CI: 24, 41%) of participants had a history of previous exposure to Q fever agent (past infection). Consumption of unpasteurized dairy products was a weak risk factor for previous exposure to *C*. *burnetii*.

**Conclusion:**

This study identified patients with acute Q fever in northwestern of Iran. The evidence from this study and previous studies conducted in different regions of Iran support this fact that Q fever is one of the important endemic zoonotic diseases in Iran and needs due attention by clinical physicians and health care system.

## Author summary

Q fever is a zoonotic contagious disease caused by a bacterium called *Coxiella burnetii*. It is mostly asymptomatic in livestock and animals. Clinical manifestations of Q fever in humans includes asymptomatic, acute and chronic to fatigue syndrome. The most frequent clinical manifestation of acute Q fever is a flu-like and self-limited illness, and clinical presentations of these patients are highly variable and extensive. Chronic Q fever is accompanied by symptoms such as endocarditis, vasculitis, prosthetic joint arthritis, osteoarticular infection and lymphadenitis. Studies conducted in Iran emphasize that Q fever is an endemic disease in different parts of Iran. Since few studies have been conducted to identify acute Q fever patients with in Iran, the present study set to investigate the prevalence of acute Q fever among at-risk individuals in northwestern Iran. The prevalence of acute Q fever was 14.0% among 116 suspected febrile patients. It was also shown that 32% of the participants had serological evidence of previous infection (past infection) with Q fever. The findings showed that most clinical symptoms in patients with acute Q fever were fever, headache, fatigue and weakness, arthralgia, myalgia, chills, chest pain and dyspnea, respectively.

## Introduction

Q fever is a zoonotic contagious disease caused by an intracellular gram-negative bacterium called *Coxiella burnetii* [1]. Q fever is mostly asymptomatic in livestock and animals, except in some cases, where it causes abortion or stillbirth. Infected animals shed this bacterium in their milk, faeces, urine and especially in birth products [2].

Inhalation of infectious aerosol particles constitutes the major route of acquiring the disease in humans, so inhalation of only one single *C*. *burnetii* can cause illness in humans [3]. Nevertheless, other routes of transmission of this infection to human are consumption of contaminated milks and dairy products, tick bites and transmission from a person to person [4]. Domestic ruminants (including cattle, sheep and goats) are the most important reservoirs of *C*. *burnetii* in the nature. However, transmission of the infection to human by dogs, cats, rabbits, birds, reptiles and arthropods, especially ticks and mites, has also been reported [5,6].

Clinical manifestations of Q fever in humans include asymptomatic, acute, and chronic to fatigue syndromes. Almost 60% of the infected people may not show any clinical symptoms. Acute Q fever is defined as a primary infection with *C*. *burnetii* [5,7]. The most frequent clinical manifestation of acute Q fever is a flu-like and self-limited illness, and the major clinical presentations of these patients are high and prolonged fever, severe headache, coughing, atypical pneumonia, hepatitis, myalgia, arthralgia, cardiac involvement, skin rash and neurologic signs [2,8]. The case fatality rate of acute Q fever is reported 1–2% [4,5]. Chronic Q fever is a disease occurring in less than 5% of acutely infected patients. It may occur several months, years, or even decades after the onset of the acute infection. This form of the disease can occur after infection with or without symptoms. Chronic Q fever is accompanied by symptoms such as endocarditis, vasculitis, prosthetic joint arthritis, osteoarticular infection and lymphadenitis.[7,9]. Endocarditis and vascular infection caused by Q fever are fatal if untreated [10].

Q fever is mainly diagnosed based on serological tests and antibody patterns that are different between acute, convalescent and chronic forms of the disease. There are two distinct antigenic phases to which humans develop antibody responses. Acute or chronic form of Q fever is diagnosed based on the dominant type of antibodies in response to antigens of phase I and II [11]. In acute Q fever infection, antibodies against phase II antigens are predominant, whereas phase I antibody titers are more prevalent in cases of chronic Q fever [10,12,13].

In Iran, the first clinical cases of acute Q fever were reported in 1952, including two patients with symptoms of severe fever and neurological signs in Abadan city, southwest Iran [14]. Furthermore, in 1970, four acute Q fever patients with pneumonic illness were reported from Shiraz, southern Iran [15]. Forty nine patients with acute Q fever were reported from Abadan city during 1970 to 1973 [16]. From 1972 to 1976, 80 patients with acute Q fever were diagnosed, among them three cases had pleuropericarditis lesions [17]. From 1976, the disease was neglected in Iran, and no human case was reported. In 2009, *C*. *burnetii* antibodies were reported in febrile patients in Kerman Province, southeastern Iran, and investigation on Q fever was resumed [18]. Afterwards, various seroepidemiological studies were conducted on animal and human population [19–23]. The first patient with Q fever endocarditis was reported in 2013 in Tehran [24]. Studies conducted in Iran emphasize that Q fever is an endemic disease in different parts of the Iran [25].

Since a few studies have been conducted to identify patients with acute Q fever in Iran, present study aimed to investigate the prevalence of acute Q fever among at-risk individuals in northwestern Iran.

## Materials and methods

### Study area

Tabriz is one of the major cities in Iran and the capital of East Azerbaijan Province located in northwestern Iran. This city, 237 square kilometers, is the third-largest city in Iran. The population of Tabriz and its suburb is approximately 1.8 million. East Azerbaijan Province is among the top five provinces in Iran in terms of production of dairy products with 10 million animal units. Tabriz has a semi-arid climate with hot summers and cold winters. Due to its mountainous climate, keeping and breeding livestock, especially sheep and goats are very common in this province. Dairy sheep and goats have an important role in dairy industry, and their milk is usually used to produce various traditional cheeses such as Lighvan cheese.

### Study design

The study was carried out in 2013 in Tabriz County. Individuals (1) with high-risk occupation exposed to animals or livestock products or samples of human patients (veterinarians, farmers, butchers and laboratory personals), (2) people living in areas close to livestock whereabouts, or (3) people with a history of keeping animals (including livestock and pets) in the previous two months, were enrolled in the study provided that they had a nonspecific febrile illness (fever above 38°C accompanied by symptoms such as fatigue, myalgia, chills, headaches, atypical pneumonia and dyspnea). Sampling was done randomly among patients who referred to the Central Laboratory of East Azerbaijan Province (in Tabriz city) and had the above criteria.

### Ethical considerations

The ethical committee of the Pasteur Institute of Iran approved the consent procedure, the proposal and the protocol of this study, covering all the samples (blood), questionnaire and verbal informed consent as most participants were either illiterate or had a primary education.

### Sampling

After obtaining informed consent from participants, researcher-developed questionnaire including demographic characteristics and Q fever risk factors were collected from each person by a researcher-developed questionnaire. Then, 6-ml blood sample was taken from each patient, and a second blood sample was taken after 4 weeks. Blood samples were centrifuged for 10 minutes at 3000 rpm and were kept at -20°C after extraction of their sera. Sera samples were transferred to the national reference laboratory of Plague, Tularemia and Q fever (Research Centre for Emerging and Reemerging Infectious Diseases, Pasteur Institute of Iran).

### Serological tests

IgG phase II antibodies against *C*. *burnetii* were detected using a commercial quantitative enzyme-linked immunosorbent assay (ELISA) kit (Serion ELISA classic, Institut Virion/Serion GmbH, Würzburg, Germany) and according to the manufacturer's instructions. Paired sera samples of each patient were tested simultaneously. Dilution protocols were used according to the manufacturer's instructions, using a 1:500 dilution for the IgG phase II assay. The plates were read at 405 nm using a microplate reader (ELx808, BioTek Instruments Inc., USA). Obtained ODs were analyzed according to the Virion/Serion protocol and IgG phase II was quantitatively reported. IgG phase II extinctions were expressed in U/ml titer using a logistic-log-model calculation and were defined as positive when the titer was >30 U/ml, as borderline when the titer was 20–30 U/ml and as negative when the titer was < 20 U/ml. The laboratory diagnosis of acute infection by *C*. *burnetii* was made based on any of the following serological criteria: (1) seroconversion, defined as the appearance of specific antibodies against the phase II antigens of *C*. *burnetii* at a titer of at least 30 U/ml in the convalescent phase (whereas the serum antibody titer was negative at the initial acute phase), and (2) a fourfold increase in serum antibody titer between the acute phase and the convalescent phase (in two blood samples obtained 4 weeks apart). If the primary and the secondary serum titers were positive, and the fourfold rise was not observed in antibody titers, it was considered as a previous history of exposure to Q fever (past infection).

### Statistical analysis

Statistical analysis was performed using the STATA software version 11. Descriptive data were reported in numbers (percentage). Chi-square test was used to assess the association among the categorical variables. P-values less than 0.05 were considered statistically significant, and P-values between 0.05 and 0.1 were considered as borderline significant.

## Results

A total of 140 patients were initially enrolled; the second blood sample was taken from 116 patients (82.9%). Mean (SD) age of the patients was 39.3 (18.05) years; 61.4% of them were male. 28.6% were urban residents and 47% had high-risk occupations for Q fever infection; 81.2% of patients had a history of domestic animal keeping, and 93.2% lived near animal shelters. Histories of unpasteurized dairy product's consumption, abortion (among females), and tick bites were reported in 62.3%, 12.2%, and 4.7%, respectively.

The prevalence of acute Q fever was 13.8% (95% CI: 8.0, 21.0%). In terms of acute Q fever infection, there was no statistically significant difference between people were exposed to risk factors (including gender, age, living location, history of residence in nearby animal shelters, previous consumption of unpasteurized dairy products, history of abortion (in women) and history of ticks bites) and those were not (Table 1).

**Table 1:** Frequency (%) of risk factors in acute Q fever patient and a history of infection with Q fever.

Variables		Total (% acute Q fever infection) n = 116	P-value	Total (% history of infection with Q fever) n = 124	P-value
**Gender**					
	Male	72 (11.1)	0.28	78 (32.1)	0.94
	Female	44 (18.2)		46 (31.6)	
**Age**					
	Under 20	25 (16)	0.56	22 (31.8)	0.97
	20–40	37 (16.2)		38 (31.6)	
	40–60	42 (14.2)		49 (34.7)	
	Over 60	11 (0)		14 (28.6)	
**Living location**					
	Urban	33 (12.1)	0.67	35 (28.6)	0.55
	Rural	79 (15.2)		85 (34.1)	
**High-risk occupations**					
	Yes	53 (13.2)	0.89	58 (31.1)	0.74
	No	57 (14.1)		59 (33.9)	
**Animals keeping**					
	Yes	89 (14.6)	0.97	95 (34.7)	0.27
	No	21 (14.3)		22 (22.8)	
**Living close to livestock shelters**					
	Yes	103 (13.6)	0.83	110 (34.5)	0.20
	No	6 (16.7)		8 (12.5)	
**Consumption of unpasteurized dairy products**					
	Yes	67 (17.9)	0.42	69 (40.6)	0.07
	No	34 (11.7)		39 (23.1)	
**Abortion**					
	Yes	10 (10)	0.53	11 (9.1)	0.07
	No	33 (18.1)		37 (37.8)	
**Tick bites**					
	Yes	5 (40)	0.06	4 (25)	0.77
	No	102 (11.8)		110 (31.8)	
**Contact with newborn animals**					
	Yes	12 (8.3)	0.58	13 (15.3)	0.16
	No	92 (14.1)		98 (34.7)	

**Fig 1:**
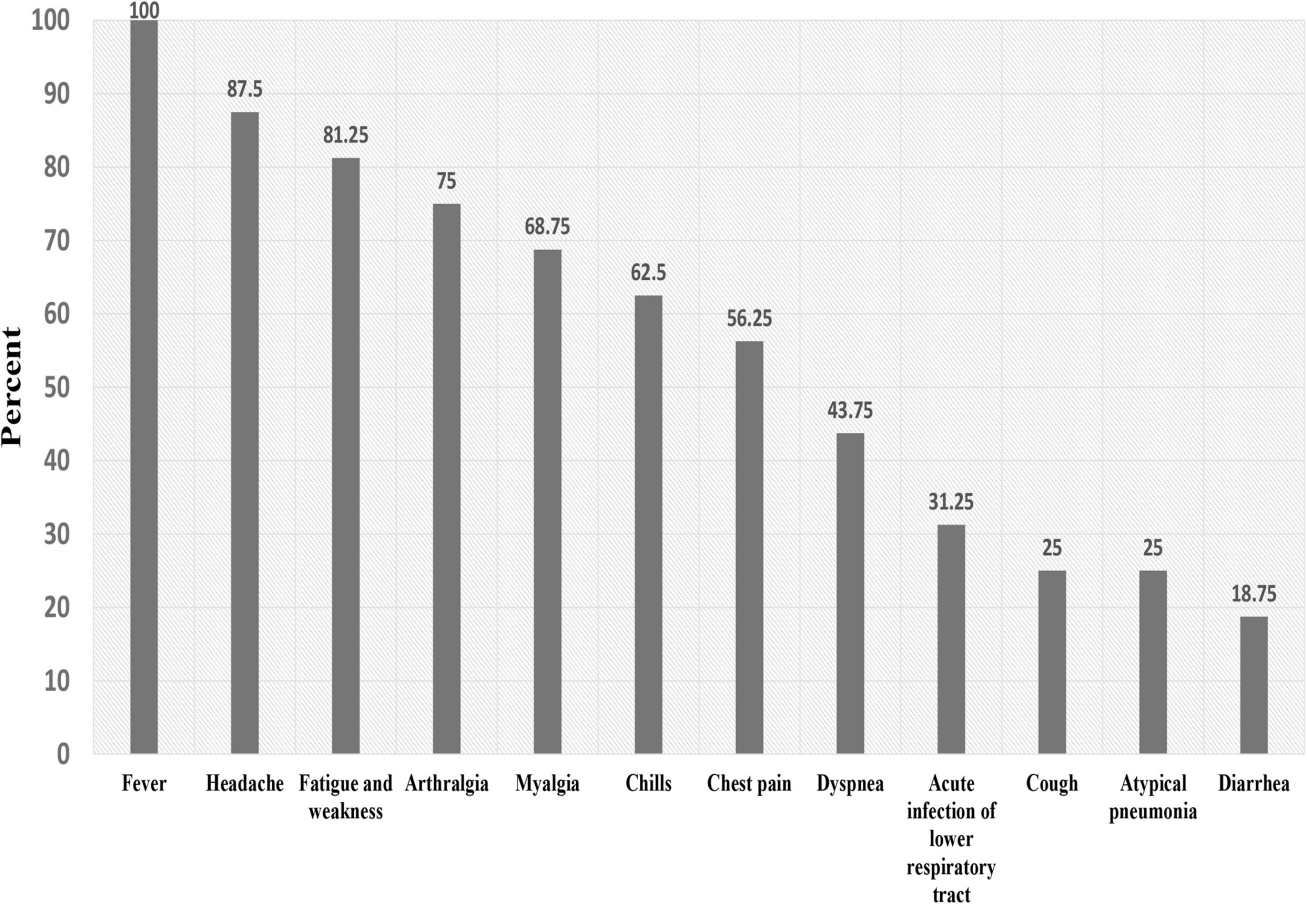
Clinical characteristics of patients with acute Q fever.

Acute lower respiratory tract infection (26.3%) and chills (20.4%) were associated with acute Q fever (borderline significance: P = 0.08). There was no significant statistical association between acute Q fever infection and other clinical symptoms (headache in the past two weeks, cough, fatigue and weakness, diarrhea, myalgia, arthralgia, chest pain, atypical pneumonia, dyspnea and hepatitis) (Table 2). Headache (87.5%) and fatigue and weakness (81.3%) were the dominant clinical characteristics among the acute Q fever patients (Fig 1). The levels of IgG phase II antibody in patients with acute Q fever are shown in Table 3.

**Table 2:** Frequency of acute respiratory infection symptoms in persons with acute Q fever infection.

Variables		Number of patients (% having acute Q fever patients)	P-value
**Headache**			
	Yes	108 (13)	0.34
	NO	8 (25)	
**Chill**			
	Yes	49 (20.4)	0.08
	NO	67 (9)	
**Chest pain**			
	Yes	64 (14.1)	0.92
	NO	52 (13.4)	
**Cough**			
	Yes	34 (11.7)	0.68
	NO	82 (14.6)	
**Fatigue and weakness**			
	Yes	97 (13.4)	0.78
	NO	19 (15.7)	
**Diarrhea**			
	Yes	10 (30)	0.12
	NO	106 (12.3)	
**Acute lower respiratory tract infection**			
	Yes	19 (26.3)	0.08
	NO	97 (11.3)	
**Arthralgia**			
	Yes	88 (12.5)	0.47
	NO	28 (17.9)	
**Myalgia**			
	Yes	96 (12.5)	0.37
	NO	20 (20)	
**Atypical pneumonia**			
	Yes	49 (8.1)	0.13
	NO	67 (18)	
**Dyspnea**			
	Yes	45 (15.5)	0.67
	NO	71 (12.7)	
**Hepatitis**			
	Yes	1 (0)	0.68
	NO	115 (14)	

**Table 3:** Levels of IgG phase II antibody titers in patients with acute Q fever.

No.	Concentration of IgG Phase II (U/ml) in first sample	Concentration of IgG Phase II (U/ml) in the second sample
**1**	5	38
**2**	24	50
**3**	26	95
**4**	16	50
**5**	5	30
**6**	6	50
**7**	12	30
**8**	13	50
**9**	11	150
**10**	15	42
**11**	<5	26
**12**	8	26
**13**	10	24
**14**	14	38
**15**	12	27
**16**	<5	500<

After excluding acute cases from all participants, 32% (95% CI: 24, 41%) of participants had a history of previous exposure to Q fever (past infection). Consumption of unpasteurized dairy products (40.6%) was a risk factor for previous exposure to *C*. *burnetii* (borderline significance: P = 0.07). None of other risk factors were associated with previous exposure to *C*. *burnetii* (Table 1).

## Discussion

This study was conducted to identify patients with acute Q fever in northwestern Iran. One hundred and forty suspected patients were enrolled in the study, 116 of whom were assessed for acute Q fever. The prevalence of acute Q fever was 13.9% among the suspected febrile patients. It was also shown that 32% of the participants had serological evidence of previous infection (past infection) to Q fever. In a similar study in Zahedan city (southeastern Iran) in 2011, 35.2% of 105 suspected feverish patients were diagnosed acute Q fever [26], which is much higher than the current report. Furthermore, in another study conducted among febrile patients suspected to have brucellosis in Kerman Province (southern Iran), 36% had phase II IgG antibody of Q fever [18]. In a study conducted in 2012 in Ardebil Province (northwestern Iran neighboring East Azerbaijan Province), remarkable seroprevalence of Q fever (33.6%) was observed among sheep [19]. The evidences from this study and previous studies conducted in different regions of Iran support the fact that Q fever is a prominent endemic zoonotic disease in Iran and needs more attention by physicians and health care system.

In similar studies conducted in France, Denmark, Mali and Croatia 2.1% (of 179794), 2.3% (of 1613), 3.9% (of 165) and 5.8% (of 552) of febrile suspected patients were diagnosed to have acute Q fever, respectively [27–30]. The rate of infection in this study was reported much higher than the so-called as in the current study, patients with epidemiological risk factors (having high-risk occupation or living in areas close to livestock or having a history of keeping animals) and clinical risk factors (having a nonspecific febrile illness) were enrolled as the cases and which increased the chance of finding acute Q fever patients. In one study, 1985 to 2009 in France, 3723 (2.1%) of 179,794 suspected patients with Q fever were diagnosed with acute Q fever, and the number of diagnosed Q fever patients was ascending over the years [28]. It could be due to the thereby improvement and development of diagnostic tests of Q fever as well as increasing attention of the physicians and the health care system to Q fever in France.

Acute Q fever was not found significantly associated with any of the studied risk factors. In a study in southeastern Iran, contacts with domestic animals and consumption of unpasteurized dairy products were identified as risk factors [26]. In two studies conducted in Australia, France and Croatia, age and gender were reported as risk factors for acute Q fever [28,30,31]. In Mali, gender, consumptions of unpasteurized dairy products and contact with newborn animals were risk factors [29]. Probably small number of patients and small number of diagnosed acute Q fever cases compared with other study are the causes for lack of finding risk factors. A larger number of suspected patients are recommended to be evaluated in future studies. Furthermore, if there had been a data bank of patients with Q fever, it would have contributed to the achievement of risk factors of Q fever infections as well as the incidence of the disease in Iran.

In this study, chills and acute lower respiratory tract infection were poorly associated with acute Q fever infection. In a study from 1983 to 1999 in Spain and in a study from 2004 to 2007 in Taiwan, the most frequent clinical pictures of Q fever patients were fever with chills [32,33]. During an outbreak of acute Q fever in the Netherlands, acute lower respiratory tract infection was the most frequent symptoms among the patients [34].

The findings showed that headache (87.5%), fatigue and weakness (81.3%), arthralgia (75%), myalgia (68.8%), chills (62%), chest pain (56.3%) and dyspnea (43.8%) were the most prevalent clinical symptoms in patients with acute Q fever. In the similar study in southeastern Iran, major clinical symptoms in patients with acute Q fever were fever (100%), myalgia (59.4%), headaches (43.2%), and arthralgia (37.8) [26]. Fever (69%) and headaches (52%) were the most prevalent clinical symptoms in patients with acute Q fever in Mali [29]. In Portugal, the major clinical symptoms were fever (100%), myalgia (68.8%), headache (62.5%), weakness (56.3%), sweating (53.1%) and chills (43.8%) [35]. In South Korea, fever (89.3%), myalgia (67.9%), weakness (53.6%) and chills (50%) were reported as the most prevalent symptoms [36]. In Spain, the most common symptoms of acute Q fever were headache (58.5%), hepatitis (49.2%), arthromyalgia (37.7%), fever (31.7%) and pneumonia (19.1%) [37]. In Taiwan, fever (99%), relative bradycardia (73%), chills (69%) and headaches (45%) were the most common clinical symptoms [33]. In Tunisia, the highest clinical signs and symptoms in patients with acute Q fever were fever (100%), fatigue (76%), hepatitis (71.5%), chills (47.5%), headache (42.8%) and sweating (33.3%) [38]. Comparing the clinical symptoms obtained in the present study with the so-called studies, the most non-specific symptoms for acute Q fever include fever, headache, chills, myalgia, arthralgia, weakness and fatigue and there are little differences in clinical symptoms. Although, some clinical signs such as hepatitis have a highlighted role in some countries[37,38], they were not observed in the current our study and some other studies. If this disease is a part of the health surveillance system in Iran and also if more clinical cases of Q fever are diagnosed and recorded, a much better view of clinical symptoms of Q fever can be obtained patients data.

In this study, 32% of participants showed evidence of previous infection (past infection) to Q fever; this rate was less than one in Zahedan city (34.3%) [26] and Kerman Province (36%) [18]. In similar studies, 35.5% in Mali [29], 21.74% in Croatia [30] and 8.7% [27] in Denmark were previously infected with Q fever. In a systematic review conducted in Africa, human seroprevalence was <8% with the exception of studies among children and in Egypt, it was 10–32% [39]. In the seroepidemiological studies of Q fever among various populations in Iran, different rates have been also reported; seroprevalence of Q fever was reported 22.5% in the Sistan va Baluchestan Province among butchers and slaughterhouse workers [40], 27.8% in the Kurdistan Province (western Iran) among butchers, slaughterhouse workers, hunters, health care workers, and patients who referred to medical laboratory [21] and 68% in Kerman Province among slaughterhouse workers [22]. Seroprevalence of Q fever was reported 12.8% in Northern Ireland [41] and 3.1% in USA [42], compared with the lower compared with the studies carried out in Iran.

The consumption of unpasteurized dairy products was a weak risk factor for previous exposure to *C*. *burnetii*. *C*. *burnetii* were isolated from livestock milks (bovine, ovine, caprine, and camel) in the different parts of Iran [43–46]. Therefore, the risk factors must be considered seriously.

Among various diagnostic methods of Q fever, serological methods are the gold standard method for diagnosis. There are a variety of serological methods for the detection of *C*. *burnetii* antibodies such as complement fixation assay (CFA), ELISA and indirect immunofluorescence assay (IFA). Although the reference method for serological diagnosis of Q fever is IFA, ELISA was used for serological diagnosis of Q fever due to lack of access to IFA. In a very comprehensive recent study, different serological methods and commercial kits were assessed in 10 different laboratories in the Netherlands and three international reference laboratories in three countries (USA, France and Australia). It was shown that IFA, ELISA, and CFA are all suitable serodiagnostic assays to diagnose acute Q fever. Sensitivity and specificity of the ELISA methods in the detection and diagnosis of IgG phase II antibody compared with reference method (IFA) were 100 and 100, respectively. Therefore, ELISA can be used as an alternative method for the diagnosis and screening of Q fever particularly in cases of acute Q fever [47].

A larger number of patients are recommended to be studied for better understanding of Q-fever risk factors. Also, molecular diagnostic techniques are recommended to be used along with serological methods.

## Supporting information

S1 Table STROBE checklist. (DOCX)
